# LSVT-BIG Improves UPDRS III Scores at 4 Weeks in Parkinson's Disease Patients with Wearing Off: A Prospective, Open-Label Study

**DOI:** 10.1155/2017/8130140

**Published:** 2017-02-01

**Authors:** Tatsuya Ueno, Megumi Sasaki, Haruo Nishijima, Yukihisa Funamizu, Tomoya Kon, Rie Haga, Akira Arai, Chieko Suzuki, Jin-ichi Nunomura, Masayuki Baba, Masahiko Tomiyama

**Affiliations:** ^1^Department of Neurology, Aomori Prefectural Central Hospital, Aomori, Japan; ^2^Department of Rehabilitation, Aomori Prefectural Central Hospital, Aomori, Japan

## Abstract

The efficacy of LSVT-BIG for advanced Parkinson's disease (PD) patients with wearing off remains to be determined. Therefore, we evaluated whether LSVT-BIG improves motor disability in eight PD patients with wearing off. Unified Parkinson's Disease Rating Scale (UPDRS) scores, daily off time, and mobility assessments were evaluated during the “on” time before and after the LSVT-BIG course. LSVT-BIG significantly improved UPDRS III scores at 4 weeks and UPDRS II scores in the “off” state at 12 weeks, with no changes in the other measures. The findings suggest that LSVT-BIG may be an effective therapy for advanced PD patients with wearing off.

## 1. Introduction

Wearing off is a gradual decrease in the duration of effect of each dose of medication for Parkinson's disease (PD) [1], which leads to a reduced quality of life in patients with PD [2]. Dopamine replacement therapy alone cannot satisfactorily treat patients with advanced PD with motor fluctuations, such as those resulting from wearing off [3, 4]. Deep brain stimulation is considered for severe wearing off and troublesome dyskinesia; however, it has been reported that axial symptoms affecting speech, gait, and postural instability progressively worsened 5 years after surgery [5]. Therefore, an alternative procedure, such as exercise, is needed for the treatment of advanced PD with wearing off.

Exercise is an established adjunctive therapy in PD. Lee Silverman Voice Treatment- (LSVT-) BIG is an exercise course that focuses on intensive high-amplitude movements to restore normal movement amplitude by recalibrating the patient's perception of movement [6] and improves motor performance measured by the Unified Parkinson's Disease Rating Scale (UPDRS) III in patients with PD [7–10]. However, previous studies demonstrating the efficacy of LSVT-BIG did not address the effect on “off” time [7–10]. Therefore, the efficacy of LSVT-BIG for PD patients with wearing off remains to be determined. To our knowledge, this is the first study to evaluate whether LSVT-BIG improves motor disability and reduces daily off time in PD patients presenting with wearing off motor fluctuations. We hypothesized that LSVT-BIG treatment would improve UPDRS II and III scores, reduce daily off time, and improve performance on the 10-meter walk test and Time Up and Go (TUG) test.

## 2. Participants and Methods

Eight Japanese patients with idiopathic PD referred from our outpatient clinic were enrolled between May 2013 and March 2014. Participants were required to fulfill diagnostic criteria for idiopathic PD [11] and had a daily off time of more than 2 hours per day. Other inclusion criteria comprised Hoehn and Yahr (H-Y) Stages II–III, outpatient treatment, and stable medication 4 weeks prior to beginning the study. Exclusion criteria were as follows: patients without informed consent; unavailable self-report on/off diary (for 3 consecutive days); patients with untreated cancer, dementia (MMSE < 24), severe depression, anemia, or hypotension; abnormal liver, renal, cardiopulmonary function; or a comorbidity affecting mobility or ability to exercise. This study was conducted in accordance with the Declaration of Helsinki and was approved by the ethics committee of Aomori Prefectural Central Hospital, Japan. All participants provided informed consent.

One physiotherapist (MS) certified as an LSVT-BIG instructor delivered all BIG sessions and also provided instructions for patients. LSVT-BIG is delivered face-to-face at a treatment dosage of 4 days/week for 4 weeks (1-hour sessions). Training has previously been described in detail [6]. After the 4-week course of LSVT-BIG, participants were encouraged to exercise for 15 to 20 minutes (some parts of LSVT-BIG) regularly at home in addition to the LSVT-BIG session. They had been on a stable regimen of drugs for at least 4 weeks before the entry and during the evaluation period.

All participants reported their on/off state using the self-report on/off diary for 3 consecutive days before assessment. UPDRS III was scored by an experienced neurologist (MT) [12]. The evaluating neurologist was not blinded to the subject's participation in this study. We made an effort to pay attention to the nonspecific effect of increased attention from hospital staff. We analyzed the following measures for changes from baseline to post-LSVT-BIG (4, 8, and 12 weeks): daily off time, UPDRS parts II and III [12], 10 m walk test, and TUG test [13] during “on” periods. UPDRS III, 10 m walk test, and TUG at 4 weeks were evaluated before the final LSVT-BIG session.

Statistical analyses were performed with the freely available EZR software version 1.32 (Saitama Medical Center, Jichi Medical University, Saitama, Japan) [14]. A probability level of 5% (*P* < 0.05) was considered statistically significant. Variables are presented as median [interquartile range]. The quantitative data were evaluated to determine whether they followed a normal distribution using the Shapiro-Wilk test. UPDRS II “off” score and III “on” score were normally distributed in the Shapiro-Wilk test, and we performed a one-way ANOVA with repeated measures followed by the paired *t*-test, with Bonferroni correction for multiple comparisons. UPDRS II score (on), TUG, 10 m walk test, and daily off time were not normally distributed in the Shapiro-Wilk test (*P* < 0.05), so we performed the Friedman test followed by the Wilcoxon test, with Bonferroni correction for multiple comparisons.

## 3. Results

Clinical characteristics of the patients are listed in Table 1. All eight patients completed the LSVT-BIG program and received all evaluations. Male/female ratio was 3/5. Their mean age (SD) was 63.2 (7.2), disease duration was 12.8 (5.1) years, and levodopa equivalent daily dosage (not including trihexyphenidyl, istradefylline, and zonisamide) was 1040 (395) mg [15].

LSVT-BIG significantly improved UPDRS III “on” scores at 4 weeks compared with baseline scores (baseline median UPDRS III “on” scores [interquartile range]: 12.5 [8.8–15.5]; 4 weeks: 8.5 [6.5–11.5]; 8 weeks: 8.0 [6.0–11.0]; 12 weeks: 9.5 [7.8–13.3]) (Table 2). However, the UPDRS III “on” score increased again at 12 weeks.

The UPDRS II score during the “off” state was significantly ameliorated at 12 weeks (baseline median UPDRS II “off” scores [interquartile range]: 14.5 [10.8–18.2]; 4 weeks: 13.0 [9.0–15.3]; 8 weeks: 14.0 [7.0–15.8]; 12 weeks: 12.5 [8.3–16.0]) (Table 2).

Daily off time, UPDRS II during the “on” state, and gait performance of the 10 m walk test and TUG were not significantly different from baseline measures at 4, 8, or 12 weeks (Table 2).

## 4. Discussion

This prospective open-label study revealed two important outcomes of LSVT-BIG in PD patients with wearing off. First, LSVT-BIG improved UPDRS III scores during the “on” state throughout the training course. However, this efficacy did not last long, because the scores increased at the end of the study. Second, LSVT-BIG ameliorated the UPDRS II score during the “off” state at the end of this study.

Several studies showed that this training led to improved motor performance in PD patients [7–10]. The ameliorating effect of LSVT-BIG was higher in H-Y stage I than in H-Y stage III [7]. We showed that LSVT-BIG improved UPDRS III scores in patients with wearing off, which may provide a therapeutic option for the management of advanced PD patients. However, this efficacy did not continue for more than 4 weeks after the final LSVT-BIG session. Our results suggest that motor skill learning is maintained in advanced PD patients with wearing off, but the progressive degeneration of nigrostriatal dopaminergic neurons may result in an unsustained motor learning system. This problem may also contribute to a lack of effects of self-exercise at home [16, 17]. LSVT-BIG is delivered one-to-one with intensive motivation and feedback [6], an approach considered to be more effective than self-exercise [8]. Therefore, it appears to be well-advised to continue active intervention after the 4-week training course to maintain the improvements.

LSVT-BIG also ameliorated UPDRS part II score during the “off” state at the end of this study. There were a tendency for a decrease of UPDRS II scores at 4 weeks and 8 weeks, but statistical analysis showed no significant differences. The UPDRS II score reflects activity of daily living [12]. Indices of quality of life, such as Parkinson's Disease Questionnaire-39, were used in previous studies, but the studies did not evaluate UPDRS II scores [7–10]. Amelioration of motor performance in PD patients with wearing off may impact their activity of daily living [18]. Therefore, these results may be associated with improvement of motor performance in PD patients with wearing off.

Our findings showed that LSVT-BIG did not decrease the daily off time but improved motor performance for levodopa. Wearing off is caused by a progressive loss of nigrostriatal dopaminergic neurons and altered postsynaptic responses to dopamine [1]. Thus, we speculate that LSVT-BIG may directly or indirectly alter the postsynaptic response to dopamine in the basal ganglia by relearning normal movement.

Generalization of our findings is limited by the open-label study design and sample size. Daily off time did not significantly decrease in this study, but there was a tendency for a decrease of 1.8 hours from baseline to 4 weeks. Investigation of a larger sample size may have a different outcome. Additionally, we did not investigate the UPDRS part III score in the “off” state. This study was performed in patients attending outpatient visits, but who were not hospitalized. In PD patients with wearing off, the “on” state is required to visit hospital, so we evaluated the UPDRS part III score during the “on” state. This study showed that LSVT-BIG improved motor performance in the “on” state. More research is needed to determine if LSVT-BIG would be useful to raise the level of motor performance in “off” state patients. Finally, this study did not have a matched comparison group. An open-label noncontrolled study may show positive results due to the nonspecific effect of more intensive treatment staff contact and interaction.

In conclusion, our findings suggest that LSVT-BIG may provide a therapeutic option for the management of PD patients with wearing off. However, satisfactory amelioration may last only a short time. Accordingly, it is necessary to continue active interventions to maintain the improvements even after the LSVT-BIG course has been completed. Further studies addressing these matters are needed to confirm whether LSVT-BIG is useful for advanced PD patients with wearing off motor fluctuations.

## Figures and Tables

**Table 1:** Clinical characteristics of patients with Parkinson's disease.

Patients	Age (years)	Sex	Hoehn and Yahr	Disease duration (years)	Daily off time (hours)	UPDRS-III	Medication (daily dose)
1	61	F	3	11	3.2	14	L/C 400 mg, ROP 12 mg, AMA 150 mg
2	66	F	3	13	5	13	L/C 650 mg, PRA 1.5 mg, AMA 300 mg, SEL 2.5 mg
3	58	F	2.5	8	3.2	20	L/C 300 mg, CAB 3 mg, AMA 150 mg
4	72	M	2	11	5.2	0	L/B 700 mg, ENT 600 mg, PRA-CR 4.5 mg, PER 750 *μ*g, SEL 5 mg, ZON 25 mg, IST 20 mg
5	53	M	2	13	5.2	8	L/C 600 mg, CAB 2 mg, AMA 150 mg, ZON 25 mg, IST 40 mg, TRI 6 mg
6	71	F	2	8	6	12	L/C 600 mg, PRA 1.5 mg, AMA 150 mg, IST 20 mg
7	69	M	3	24	4.8	9	L/C 350 mg, PER 1500 *μ*g, ENT 300 mg, AMA 100 mg, ZON 25 mg, IST 40 mg
8	56	F	3	14	4.9	26	L/C 1000 mg, PRA-CR 4.5 mg, AMA 300 mg, ZON 50 mg, TRI 3 mg

L/C, levodopa/carbidopa; L/B, levodopa/benserazide; PRA, pramipexole; PRA-CR, pramipexole continuous release; ROP, ropinirole; CAB, cabergoline; AMA, amantadine; SEL, selegiline; ENT, entacapone; ZON, zonisamide; IST, istradefylline; TRI, trihexyphenidyl; UPDRS-III, Unified Parkinson's Disease Rating Scale part III.

**Table 2:** Outcome measures from baseline to 12 weeks.

	Baseline	4 weeks	8 weeks	12 weeks
UPDRS II (off)	14.5 [10.8–18.2]	13.0 [9.0–15.3]	14.0 [7.0–15.8]	12.5 [8.3–16.0]^*∗*^
UPDRS II (on)	0.5 [0–2.5]	0 [0–1.3]	0 [0–1.5]	0.5 [0–2.3]
UPDRS III (on)	12.5 [8.8–15.5]	8.5 [6.5–11.5]^†^	8.0 [6.0–11.0]	9.5 [7.8–13.3]
Daily off time (hours)	5.0 [4.4–5.2]	3.2 [1.8–3.6]	3.7 [2.8–4.3]	3.8 [3.5–4.6]
TUG (sec)	7.8 [7.3–8.1]	6.7 [5.9–7.4]	7.5 [6.4–8.9]	7.0 [6.6–8.2]
Timed 10 m (sec)	8.7 [7.4–9.7]	8.3 [7.3–9.1]	8.0 [7.0–8.5]	8.2 [8.0–8.4]

Data are median [interquartile range]. ^*∗*^Baseline versus 12 weeks: *P* < 0.05; ^†^baseline versus 4 weeks: *P* < 0.05.

UPDRS- III, Unified Parkinson's Disease Rating Scale part III; TUG, Timed Up and Go.
